# Children Can Learn New Facts Equally Well From Interactive Media Versus Face to Face Instruction

**DOI:** 10.3389/fpsyg.2016.01603

**Published:** 2016-10-25

**Authors:** Kristine Kwok, Siba Ghrear, Vivian Li, Taeh Haddock, Patrick Coleman, Susan A. J. Birch

**Affiliations:** Department of Psychology, University of British ColumbiaVancouver, BC, Canada

**Keywords:** child development, children’s learning, interactive technology, learning and memory, cognitive development, early childhood education, research methods

## Abstract

Today’s children have more opportunities than ever before to learn from interactive technology, yet experimental research assessing the efficacy of children’s learning from interactive media in comparison to traditional learning approaches is still quite scarce. Moreover, little work has examined the efficacy of using touch-screen devices for research purposes. The current study compared children’s rate of learning factual information about animals during a face-to-face instruction from an adult female researcher versus an analogous instruction from an interactive device. Eighty-six children ages 4 through 8 years (64% male) completed the learning task in either the Face-to-Face condition (*n* = 43) or the Interactive Media condition (*n* = 43). In the Learning Phase of the experiment, which was presented as a game, children were taught novel facts about animals without being told that their memory of the facts would be tested. The facts were taught to the children either by an adult female researcher (Face-to-Face condition) or from a pre-recorded female voice represented by a cartoon Llama (Interactive Media condition). In the Testing Phase of the experiment that immediately followed, children’s memory for the taught facts was tested using a 4-option forced-choice paradigm. Children’s rate of learning was significantly above chance in both conditions and a comparison of the rates of learning across the two conditions revealed no significant differences. Learning significantly improved from age 4 to age 8, however, even the preschool-aged children performed significantly above chance, and their performance did not differ between conditions. These results suggest that, interactive media can be equally as effective as one-on-one instruction, at least under certain conditions. Moreover, these results offer support for the validity of using interactive technology to collect data for research purposes. We discuss the implications of these results for children’s learning from interactive media, parental attitudes about interactive technology, and research methods.

## Introduction

It is staggering to imagine that there are as many mobile devices in use today as there are people in the world. There are over 9.6 billion devices in use today versus 7.4 billion people currently on Earth (Radicati, 2014). Moreover, projections suggest that by the end of 2018 the number of worldwide mobile users is expected to surpass 6.2 billion. That is, roughly 84% of the world’s population will be using mobile technology by year-end 2018 (Radicati, 2014). The recent rise in the use of mobile devices is also reflected in the fact that in many developed countries the majority of parents allow their children to use them at home (e.g., Beauchamp and Hillier, 2014). Indeed, the Rideout et al. (2003) reported that, even at that time, nearly 48% of US children 6 years of age had used a computer and more than 30% had also played video games. Remarkably, US children 6 years of age and under spent, on average, the same amount of time with screen media per day (1 h, 58 min) as they did playing outside (2 h, 1 min; Rideout et al., 2003).

Due to the increasing use of mobile devices at home there has been an explosion in electronic media directly targeting young children. Apple Inc., for instance, has recognized children’s increasing mobile device use in their launch of a Kids App Store. The creation of this new category acknowledges children’s interest in using apps and recognizes that children make up a substantial portion of app users. In fact, children are the targets of over 80% of the top selling paid apps in the education category of the iTunes store (Shuler, 2012).

Despite the rapid growth in children’s use of interactive technology, research comparing the efficacy of children’s learning from interactive media versus more traditional learning contexts is still relatively scarce. The primary goal of the research presented here is to help fill that gap by experimentally comparing how well preschool-age and early school-age children learn new facts from interactive technology versus from a face-to-face interaction with an adult. In the current literature, there are studies that compare children’s learning between live interactions and video (e.g., Reiser et al., 1984; Kuhl et al., 2003; Krcmar et al., 2007; Roseberry et al., 2010) and between live interactions, video chat, and video (e.g., Roseberry et al., 2014), with results suggesting that children do not learn some types of information (e.g., language input) from TV or videos as well as they do from live interactions. For instance, research by Patricia Kuhl and her colleagues (Kuhl et al., 2003) found that between 9 and 10 months of age infants show phonetic learning from live, but not prerecorded, exposure to a foreign language. The results of this study suggest a learning process that is enhanced by social (face-to-face) interactions. On the other hand, results from a meta-analysis suggest that individuals in online learning conditions (e.g., print-based correspondence education, broadcast TV or radio, videoconferencing, stand-alone educational software) performed *better* than those receiving face-to-face instruction (e.g., in-person lectures, holding meetings with groups of students), with the important caveat that this meta-analyses included much older participants (i.e., Kindergarten through grade 12) and a variety of different learning mediums (Means et al., 2009).

Critically, empirical research on how *interactive* touch-screen devices affect learning outcomes remains extremely scarce (see Haßler et al., 2015; Radesky et al., 2015). A handful of recent studies have shown the positive effects of touch-screen mobile devices on children’s learning in a few domains. For example, Neumann (2016) found evidence to suggest a positive association between 2- to 4-year-olds’ use of touch-screen devices and their print awareness, print knowledge, and sound knowledge, suggesting that these pre-writing activities can promote the development of reading and writing skills. The use of an iPad also allows 2- to 3-year-old children to produce more continuous and complex mark making (a foundational skill for writing) when compared to the use of traditional paper and paint (Price et al., 2015). Importantly, touch-screen devices have also been shown to allow learning to transfer from the device to a physical version of a similar task (i.e., puzzles) (Huber et al., 2016). These aforementioned studies on the effects of interactive media on learning have focused on what children can learn from such devices *on their own*. However, it is important to recognize that children often engage with interactive media in a social context (e.g., in the presence of a caregiver or peer) that can influence learning. For example, research by McPake et al. (2013) showed that touch-screen devices have the potential to facilitate communicative and creative skills when the child observes an adult using that technology before trying it out on their own. It also appears that there are strategies that parents can use to enhance children’s learning when using interactive media. Research by Flynn and Richert (2015) showed that using novel interactive media allowed children to perform better on letter and number recognition and device knowledge when parents focused on the content of what was being learned, rather than focusing on the device itself. Therefore, when evaluating the efficacy of any learning approach it is important to consider the broader social context, including the level of parental and teacher involvement as well as the parents’ and teacher’s beliefs about its efficacy.

If you look at how pervasive interactive media is today in both the home and the classroom it is tempting to assume that many parents and educators believe they are effective learning tools. For example, even parents of children between the ages of 6 and 24 months report they frequently give their children a mobile device to play with (see Bedford et al., 2016; Wooldridge, 2016). Unfortunately, when asked about their reasons for giving such devices to their children, parents’ top three reasons did not include teaching and learning, but instead were to ‘entertain,’ ‘videochat,’ or ‘calm their children’ (Wooldridge, 2016). Similarly, a study by Beauchamp and Hillier (2014) reported that the most popular reason parents gave for using interactive tablets with their children was for entertainment purposes, whereas only 19% reported using them for their children’s learning. Yet, the same report revealed that 83% of these parents believed that technology is important to their child’s success in school (Beauchamp and Hillier, 2014). Parental attitudes toward interactive technology suggest that they believe their value lies primarily in entertainment, rather than in its educational potential. Indeed, a majority of parents believe that any learning from touch-screen devices is inferior to that acquired through real-world experiences and interactions (Wooldridge, 2016)^1^. Are parents’ concerns about the educational value of such devices justified? Or, are they simply due to a lack of evidence on the positive benefits of learning from interactive devices?

Despite the scarcity of rigorous experimental research on learning from interactive media, the market for children’s educational apps continues to grow, and for seemingly good reasons. On its face, interactive media has significant advantages over traditional toys and over other forms of media such as television or video including reactivity, interactivity, tailor-ability, progressiveness (i.e., the ability to become increasingly more challenging over time), and portability (Christakis, 2014). For instance, interactive screens are predetermined (like a video) but still reactive to the child’s actions (like a socially contingent interaction). In addition, the mobility of devices allows learning to happen anytime and anywhere. The student is no longer restricted to having to sit in a single location in front of a computer to use technology in an educational context. This accessibility and portability allows parents to introduce technology as a part of their child’s education at a very young age, and easily supplement learning outside of the typical classroom environment or person-to-person instruction.

As parents and teachers continue to incorporate mobile devices in children’s lives, the need for studying the effects of mobile interactive media in children’s learning becomes increasingly valuable. A study examining the prevalence of iPads in the classroom setting, for instance, found that early childhood educators across all programs and student income levels reported almost a twofold increase in tablet access from 2012 to 2014 (Blackwell, 2015). The American Academy of Pediatrics (2013) has updated their views to acknowledge the value of educational media in young children’s learning, and government agencies and school districts have committed large budgets to increase technology in classrooms. For example, Apple Inc. reported that there were over 10 million iPads in use in schools around the world as of 2013 (Apple.com, 2016). Yet, in a systematic review on how the use of interactive tablets affects learning outcomes among children, Haßler et al. (2015) concluded that policies established on the use of interactive tablets in children’s learning are based on little evidence, and highlight the need for more rigorous studies to understand how interactive tablets affect children’s learning.

In the current experiment, we compared children’s rate of learning (i.e., how much participants learned) during a face-to-face (one-on-one) instruction with a female adult versus their rate of learning from an interactive iPad application in the presence of a female adult. Our aim was to quantify and compare the amount of learning taking place between the two learning contexts, as well as to validate the use of interactive media as a means of collecting data from children for research purposes. In both the ‘Face-to-Face’ and ‘Interactive Media’ conditions, which were presented as games, children were taught new facts about animals. The procedures were analogous except that in the Face-to-Face condition a female adult instructor taught the child facts using printed visual aids (e.g., animal pictures), whereas in the Interactive Media condition the same information was presented on a touch-screen tablet accompanied by pre-recorded audio files of an adult female voice represented by a cartoon character. To examine the effects of interactive media on factual learning in early childhood, children 4 to 8 years of age were tested. A wealth of previous research has demonstrated that children’s learning and memory tends to improve with age (e.g., Gathercole et al., 2004; Hala et al., 2013). Given this, and the fact that this age range includes preschool-age children (ages 4 and 5) who spend most of their time in informal learning contexts (e.g., home, daycare, kindergarten) as well as school-age children (ages 6+) who have been exposed to more formal and structured learning contexts (e.g., classroom settings), we also examined age-related changes in children’s learning across the two conditions.

## Materials and Methods

### Participants

Eighty-six children between 4 and 8 years of age participated in this study. Forty-three children (*M* = 67.30 months, *SD* = 13.29; 15 females) participated in the Face-to-Face condition and were randomly assigned to learn animal facts in a specific order (out of four possible orders, or versions, described below). An additional 43 children (*M* = 66.56 months, *SD* = 12.68 months; 17 females) participated in the Interactive Media condition. To equate the two groups for age and order, a commonly used ‘matching’ technique was applied such that each participant in the Interactive Media condition was ‘matched’ with a previous participant from the Face-to-Face condition by selecting the participant closest to that individual in age (to the nearest month), and assigning that participant to the same order. This study was approved by, and carried out in accordance with the ethical standards of, the University of British Columbia’s Behavioral Research and Ethics Board with written informed parental consent for all subjects.

Children in both conditions were tested in a quiet setting in a child development lab, science museum, local preschool, or park setting. Ethnic demographics were similar in both the Face-to-Face (41.9% White, 18.6% East Asian, 11.6% South Asian, 20.9% Other) and Interactive Media (51.2% White, 16.3% East Asian, 4.7% South Asian, 14.0% Other) conditions. Nine additional children were tested, but their data were not included in the analyses: 3 due to a failure to complete the task and 6 due to experimenter error or technological problems.

### Materials

#### Learning and Testing Materials

Two sets of four trivia questions (Set A and Set B; see **Table 1** for a complete list of questions) were used in this experiment. Each child was taught the answers to one set of questions, but not the other. The ‘untaught’ questions served as a baseline measure of question difficulty and ensured that children of this age did not know the answers to these facts beforehand. We varied whether children were taught Set A questions or Set B questions as well as which set of questions came first, resulting in four possible orders or versions (i.e., Set A first and Set A taught, Set B first and Set B taught, Set A first and Set A untaught, and Set B first and Set B untaught). Participants in the Face-to-Face condition were assigned to one of the four possible versions at random. Each participant in the Interactive Media condition was assigned the version that corresponded with their closest age ‘match’ from the Face-to-Face condition to equate the groups for age, question set, and question order.

**Table 1:** List of factual questions and four possible response options used during the testing phase.

Question Set	Question	Option 1	Option 2	Option 3	Option 4
A	Which kind of insect, or bug, is the smallest?	**Fairyfly**	Leaf beetle	Lady bug	Seed bug
	Which kind of bear is the largest?	Grizzly bear	**Polar bear**	Black bear	Panda bear
	Which kind of dog cannot swim?	Pug	Brussels griffon	Poodle	**Basset hound**
	Which animal is the best jumper?	**Flea**	Goat	Grasshopper	Rabbit
B	Which kind of bird can fly the highest?	Peregrine falcon	Malleefowl	**Ruppell’s vulture**	Eagle
	Which part of the body is called the nape?	Bottom of the feet	Back of the knees	**Back of the neck**	Top of the head
	Which animal is the fastest in the sea?	Clownfish	**Sailfish**	Pilot whale	Mako shark
	Which animal has the best hearing?	Elephant	Bat	Three-toed sloth	**The Greater Wax Moth**

*Correct answers are indicated in bold text. When children were asked to choose between the four responses option, they were presented with four pictures displaying each option; none of these images were displayed during the learning phase. The position of the pictures is reflected in the table, such that Option 1 was presented in the top left quadrant, Option 2 in the top right quadrant, Option 3 in the bottom left quadrant, and Option 4 in the bottom right quadrant (see **Figure 2** for a sample)*.

#### Visual Aids and Equipment

**FIGURE 1:**
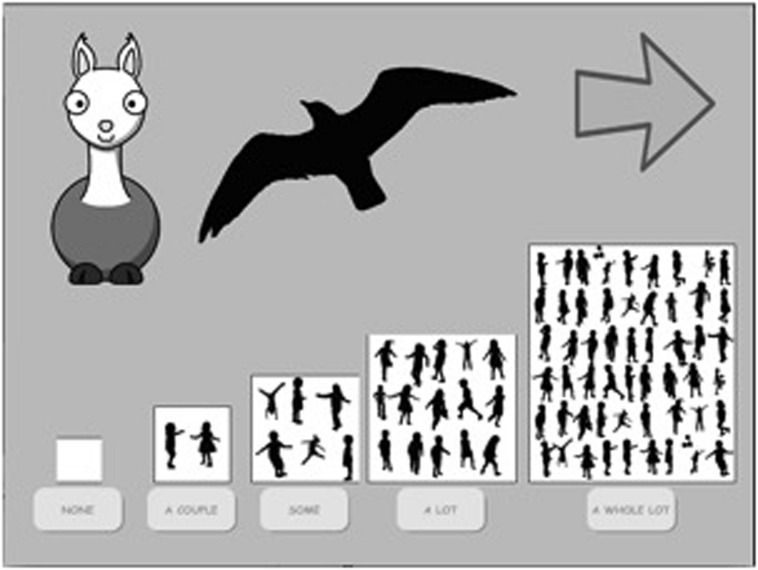
**Sample screen presented to children in the Interactive Media condition featuring a cartoon, Laila the Llama, that taught the children facts using a corresponding pre-recorded female voice**. The recordings were triggered by the child’s responses or by the lapse of a preset amount of time in such a way that they appeared ‘socially contingent’ with the child.

Each question and response option was accompanied by images as visual aids. All images in this study were publicly sourced and labeled for reuse. For the Face-to-Face condition, the images were printed out and shown by the experimenter (one of eight adult female research assistants). For the Interactive Media condition, an iPad app was developed in Swift 2.0 featuring a cartoon figure (Laila the Llama) that narrated the app, replacing the role of the live instructor (see **Figure 1**). The app was presented using an iPad Pro. The same images used in the Face-to-Face condition were used in the Interactive Media condition (see **Figure 1** for a sample). Audio recordings of the instructions, questions, and response options were recorded for the app using an adult female’s voice (a different voice from the 8 female research assistants; one of whom was always present during the experiment). A full version of the interactive media app is available on-line: http://tinyurl.com/jzk5mym.

### Procedure

Each child was tested individually in the presence of an adult female research assistant. The procedure, which was presented as a game, took place in three main phases: A Demonstration Phase, A Learning Phase, and a forced-choice Testing Phase. Prior to the Demonstration Phase, the research assistant introduced herself to the child and asked if the child would like to play a game. In the Interactive Media condition, the female researcher subsequently activated the app wherein the cartoon llama introduced herself saying, “Hi, I’m Laila the Llama! Would you like to play a game with me?” via the prerecorded audio files.

#### Demonstration Phase

If children agreed to participate in the game, the research assistant (in the Face-to-Face condition) and Laila the Llama (in the Interactive Media condition) demonstrated how to play the game, saying, “Let me show you how to play. See this board? There’s no children here, there’s a couple of children here, there’s some here, a lot here, and there’s a whole lot of children here! I am going to ask you some questions and you can show me how many children your age will know the right answer by pointing to one of these.” (See **Figure 1**). The demonstration continued with the interactive tutorial or the live instructor providing three sample questions and answers (e.g., “A cow says moo. How many children your age will know that? I think a whole lot of children will know that so you’d point here.” (i.e., point 5 on the scale). This process was repeated two more times to illustrate a much more difficult question and a question of medium difficulty (Refer to Appendix A for a detailed description of the full Demonstration Phase). This phase lasted approximately 2-3 min.

#### Learning Phase

During the Learning Phase, children were presented with the eight trivia questions each accompanied with an ‘anchor’ image. For example, children were presented with a question about which bird can fly the highest and the accompanying anchor image was a silhouette of a bird. These anchor images were presented again later when the same question was asked during the Testing Phase. For half the trials, children were taught the answers to one set of questions (e.g., they were taught Set A), whereas they were not taught the answers to the other set of questions (e.g., they were not taught Set B). In each learning trial, the new facts were embedded in the question of how many of their peers would know the answer. This ‘guessing game-like’ learning context was intended to create a naturalistic learning situation that was engaging, but not anxiety-provoking or overly formal, and as such children were not told this was a teaching lesson or that their memory for the facts would be tested.

For each taught trial, children heard the new fact, followed by the question, “How many children your age will know [question]?”. For instance, “The Ruppell’s vulture is the bird that can fly the highest. How many children your age will know which bird can fly the highest?”. For each untaught trial, children simply heard the question, “How many children your age will know [question]?” and were not provided with the answer. For example, they heard, “How many children your age will know which bird can fly the highest?”^2^. Children answered these questions by tapping on, or pointing to, one of the five buttons illustrating a different number of peers on a five-point scale (described in Appendix A in the Supplemental Materials; see also **Figure 1**). Children could also have the question repeated^3^. In the Face-to-Face condition, the instructor asked children if they would like to have the question repeated, whereas in the Interactive Media condition the Llama instructed them on how to hear the question again (e.g., “tap on me” to hear the question again). This phase lasted approximately 4 min.

#### Testing Phase

**FIGURE 2:**
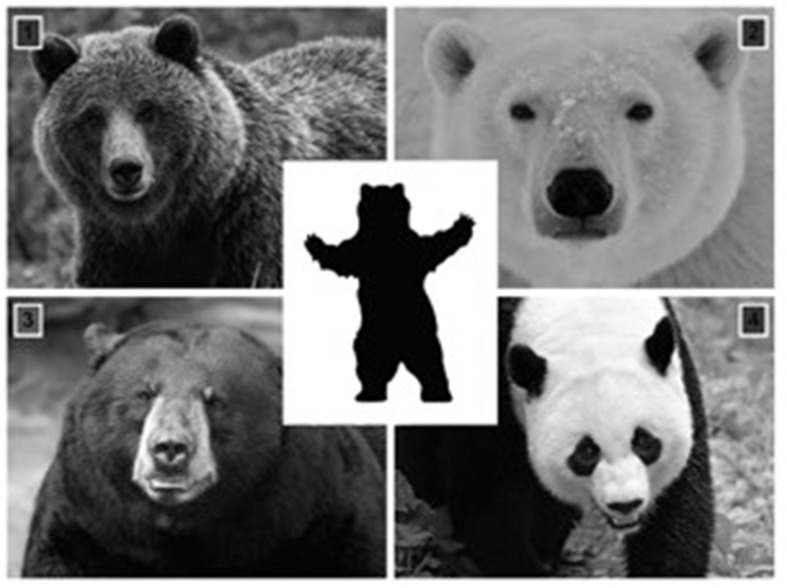
**A sample of the anchor picture (the silhouette of a bear) surrounded by four potential response options**. Children were asked to tap their answer (Interactive Media condition) or point to their response (Face-to-Face condition).

During the Testing Phase, children were presented with all eight trivia questions again in the same order as they were presented during the Learning Phase. In the testing phase, the questions were presented in a multiple-choice format with four options surrounding the ‘anchor’ images (refer to **Figure 2** for a sample and **Table 1** for the placement of the correct answers). The location for the correct answer was predetermined by pseudo random order but was fixed for all children.

The testing phase occurred without delay after the Learning Phase in the Face-to-Face condition. The Interactive Media condition began with an interactive tutorial, lasting 30 s, where Laila the Llama explained how to choose their answers using the pre-set response options and prompted children to do one practice trial (e.g. “What animal says moo? Is it a cow, a chicken, a pig, or a horse?”) while the pointer finger moved along with the audio to direct attention to the corresponding image. Importantly, the animations of the images were synced to the timing of the audio so that the audio labeled the images as they appeared; after appearing, the images were grayed out to indicate the inability to interact with the screen until the children had seen and heard all the options. The subject was prompted to tap on an image and the app waited until this action was completed before surfacing the green arrow to allow the child to move on when ready.

During test, the instructor (i.e., the female adult or Laila the Llama) asked each child the trivia questions, and presented the four options by pointing to each image and labeling it (e.g., a Peregrine falcon, a Malleefowl, a Ruppell’s vulture, or an Eagle). In the Interactive Media condition this was done through an audio recording of each question and its four options. The children indicated their responses by pointing or tapping on the intended image. Once again, children were able to get the question repeated during this Testing Phase by either asking the instructor (Face-to-Face condition) or tapping on the center ‘anchor’ image (Interactive Media condition). If a child requested a question repeat in the Interactive Media condition, all elements on the screen disappeared and re-entered in the same manner as before. The latter feature was limited to three repeats. After the eight trivia questions, the instructor thanked the child for playing the game and presented him or her with four stars. In the Face-to-Face condition, the stars were stickers (that the child kept), and in the Interactive Media condition, the stars were presented on the screen.

## Results

Children’s rate of learning across the two conditions was determined by computing the total number of taught trials (out of 4) in which they chose the correct answer. These totals served as our dependent variable.

### Preliminary Analyses

We first ruled out any order effects (i.e., which set was taught, Set A or Set B, and which set came first, Set A or Set B), *p*s > 0.10. To additionally rule out gender effects, we tested whether children showed a higher rate of learning in the Face-to-Face condition compared to the Interactive Media condition using a 2 × 2 ANOVA with gender and condition as between-subjects factors. No differences by gender were obtained, *F*(1,84) = 0.011, *p* = 0.915; therefore the remainder of the analyses collapse across gender and order.

### Primary Analyses

Children learned equally well from the interactive iPad app (*M* = 1.86 of four items) as they did from a live instructor [*M* = 2.12 items, *t*(84) = 0.907, *p* = 0.564, two-tailed, *d* = 0.2]. That is, children showed similar learning performance on this task regardless of whether they learned the facts from a female adult during an in-person interactive learning exercise or from an analogous learning exercise developed for the iPad. On average, children remembered approximately 2 of the 4 new facts that were introduced. Although their performance was not near ceiling, it significantly exceeded chance (25% or 1 of 4 items) in both the Face-to-Face, *t*(42) = 5.357, *p* < 0.001, two-tailed, *d* = 0.82, and Interactive Media, *t*(42) = 4.530, *p* < 0.001, two-tailed, *d* = 0.69, conditions.

Importantly, as a comparison, children performed below chance for the questions about facts that they were not taught, in both the Face-to-Face [*M* = 0.65, *t*(42) = -3.041, *p* = 0.004, two-tailed, *d* = -0.93] and Interactive Media conditions [*M* = 0.77, *t*(42) = -2.031, *p* = 0.049, two-tailed, *d* = 0.63]. The majority of children’s incorrect answers in this case reflected responses they were more familiar with. For instance, the majority of the children who were not taught the question, “Which kind of bird can fly the highest?” chose “eagle” as their answer, rather than the correct response “Ruppell’s vulture”. Thus, our interpretation is that the below chance performance for the untaught questions reflects the difficulty of the facts and children’s tendency to select the most familiar answer as a response strategy when answering questions for which they do not know the answers. Ultimately, the difference in the observed response patterns for taught versus untaught facts highlights the ability of both Face-to-Face and Interactive Media conditions to facilitate children’s learning.

**FIGURE 3:**
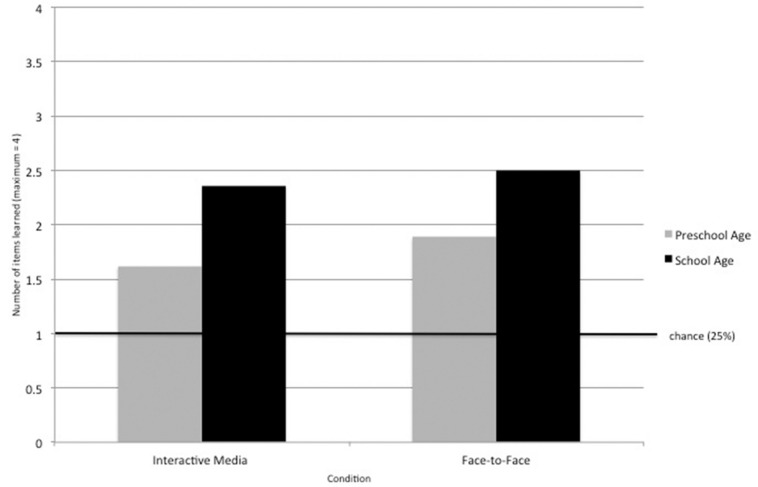
**Average number of items recalled (out of a total of 4) by Condition and Age Group**. School aged children learned significantly more items than preschool aged children. Children in both age groups recalled items at significantly greater than chance rates (25% or 1 of 4 possible answers) and there were no differences by condition.

To test whether children’s learning varied as a function of age, we examined the correlation between children’s age in months and their memory for previously taught facts. Age was positively correlated with improved memory performance, *r* = 0.341, *n* = 86, *p* = 0.001. That is, as children got older they were more likely to learn or remember the facts they had been taught. This same developmental pattern of learning was observed in both the Face-to-Face, *r* = 0.385, *n* = 43, *p* = 0.011, and Interactive Media Conditions, *r* = 0.289, *n* = 43, *p* = 0.061. Our sample includes a group of children (Preschool Age: 4 and 5 year-olds) who do not yet spend the majority of their time in formal learning contexts and a group of children who have transitioned to elementary school where they are introduced to more formal and structured learning (School Age: ages 6+). Thus, to further examine potential age differences, we split participants into School Age (age > 72 month, *n* = 30) and Preschool categories (age < 72 months, *n* = 56) and conducted a 2 × 2 ANOVA with Age and Condition as the between-subjects factors. This analysis revealed a main effect of Age, *F*(1,84) = 05.38, *p* = 0.023, with School Age children performing better than Preschool Age children, but no main effect of Condition (*p* = 0.481) and no significant interaction, *p* = 0.830. Further analyses showed that children in both age categories remembered items at above chance rates (25% or 1 of 4 items): School Age, *t*(29) = 5.787, *p* = 0.049) and Preschool Age, *t*(55) = 4.583, *p* < 0.001 (See **Figure 3**).

## Discussion

The primary goal of the present research was to compare 4- to 8-year-old children’s rate of learning factual information in an experimental learning task presented by an adult instructor versus an interactive media device (i.e., a child-friendly app designed for the iPad). Our analyses revealed that children performed equally well in the Interactive Media condition as they did in the Face-to-Face condition. That is, the 4- to 8-year-old children in our sample recalled the facts they were taught at rates significantly above chance, regardless of whether they learned those facts from an adult researcher or via an interactive iPad app in the presence of an adult researcher.

Unsurprisingly, children’s age in months was also positively correlated with their memory performance, which is consistent with a wealth of previous findings showing that children’s learning and memory improves with age (see for e.g., Roberts and Blades, 2000; Gathercole et al., 2004; Ofen et al., 2007; Hala et al., 2013). Moreover, school-age children (ages 6 through 8) performed significantly better than preschool age children (ages 4 and 5). This same developmental pattern of learning was observed in both the Face-to-Face and Interactive Media Conditions. Importantly, however, even the preschool age children performed significantly above chance. These findings suggest that face-to-face instruction and interactive touch-screen applications can be similarly effective learning methods for children ages 4 through 8.

The current study contributes to our understanding of children’s learning through interactive media, however, future research can further elucidate this process. For instance, the current study examined learning in a naturalistic, relatively informal, game-like setting where the children did not know in advance that they would be tested on the information presented. It is therefore a question for future research how interactive media compares to live instruction for more formal and explicit testing situations (e.g., where children are explicitly instructed to memorize new information for later testing). In addition, in our research the testing phase took place immediately following the learning phase. As such, it is an open question for future research whether interactive media and live instruction are equally effective for retaining newly acquired information for longer periods of time.

Given the increasingly prominent role technology is playing in children’s lives, our findings make an important contribution to a small but growing body of literature on the comparative effectiveness of so-called ‘digital learning’ versus more naturalistic or traditional pedagogical approaches. Findings from the earlier literature were somewhat mixed on the efficacy of learning from digital media. As previously mentioned, a meta-analysis examining learners from kindergarten through high school found that students in online learning conditions (e.g., correspondence learning, stand-alone educational software, broadcast TV, or radio) performed better, on average, than those in more traditional face-to-face instruction (e.g., in-person lectures, student meetings; United States Department of Education, 2009). However, in other learning contexts children’s learning is far inferior from digital media (e.g., TV) than it is from live social interactions (e.g., Kuhl et al., 2003). For instance, previous research suggests that 9 and 10 month olds show phonetic learning from live, but not prerecorded, exposure to a foreign language, and that children tend to imitate live demonstrations more than they imitate demonstrations from television, until at least 3 years of age (Zack et al., 2009).

The aforementioned *seemingly* mixed results highlight the importance of considering, and comparatively testing, the *type of media* that is used as well as the *type of learning* being tested, not to mention the age of the participants involved. Recent work by Bedford et al. (2016) made important strides in this regard. Using an online survey of 715 parents of 6- to 36-month-olds they examined how age of first touchscreen usage (retrospectively reported) related to gross motor (i.e., walking), fine motor (i.e., stacking blocks), and language (i.e., producing two-word utterances) milestones. Their results revealed that for toddlers, aged 19-36 months, age of first touchscreen use was significantly associated with fine motor skills (stacking blocks) after controlling for age, sex, mother’s education (a proxy for SES), and the age at which they achieved a fine motor milestone (pincer grip). Importantly, this effect was only present for active scrolling of the touchscreen and not for passively observing the device (e.g., video watching). No significant relationships were found between touchscreen use and either gross motor or language milestones. These data provide converging evidence with other work suggesting the potential power of digital tools to facilitate learning such as letter and number recognition (Flynn and Richert, 2015) and knowledge transfer from media learning to analogous physical problems (Huber et al., 2016; see also Semmelmann et al., 2016).

Similarly, a report by Radesky et al. (2015) reviews the limited research on the impact of interactive media use on children and suggests that interactive media can be useful for teaching concrete knowledge (e.g., science, addition, subtraction, counting, multiplication, and chemistry); however, skills such as self-regulation and empathy are perhaps best learned through interactions with peers and caregivers in naturalistic environments. Much more work is needed to investigate whether face-to-face instruction and interactive media methods are equally effective at teaching different types of information (e.g., trivia facts versus procedural information such as how to fold a flag or pitch a tent) and different kinds of skills (e.g., cognitive vs. social skills). Radesky et al. (2015) also acknowledged that interactive media can promote learning by demonstrating ideas for parent-child activities, or by modeling teaching strategies (e.g., dialogic reading, phonetic, or sound blending skills).

As mentioned in the introduction, many parents and educators hold negative attitudes toward interactive devices for learning purposes compared to the perceived benefits of ‘real-world’ learning opportunities (Wooldridge, 2016). These perceptions might lead some individuals to expect superior learning in the Face-to-Face condition. In contrast, our finding suggests that perhaps caregivers and educators do not need to be overly concerned about the use of technology for learning, given that interactive media appears equally effective as face-to-face instruction, at least for certain learning contexts (e.g., the factual learning tested in the present research). Of course, the potential benefits of children’s use of interactive technology will ultimately depend on what children are doing on the interactive device (e.g., whether the apps being used include an educational component—intentionally or otherwise).

Importantly, although facts taught in the Interactive Media condition came from a pre-recorded (albeit programmed to be interactive) voice as opposed to a live instructor in the Face-to-Face condition, an adult research assistant was always present with the child during testing and watched the child interact with the iPad game. Although the researcher did not by default say anything during the child’s interaction with the iPad, if the child got distracted she encouraged the children to keep playing, or if the child was very delayed in responding she reminded the child that they could tap on the llama to hear the question again (akin to the same kinds of encouragement offered in the Face-to-Face condition). These conditions arguably provided some social scaffolding and may have included important attentional or pedagogical cues that facilitated learning. Consistent with this notion, previous work has demonstrated that even at 12 and 15 months of age, word learning is not facilitated by repeated viewings of educational DVDs (i.e., Baby Einstein) (Robb et al., 2009). However, watching similar programs (i.e., Baby Mozart) alongside a caregiver, who scaffolded their viewing behavior and increased shared attention and turn-taking, was associated with better responsiveness and attention to the learning source (Barr et al., 2008; Fidler et al., 2010).

Similarly, although some research suggests that until around age 3 children have difficulty transferring ‘2D learning’ into the real 3D world; a so-called ‘video deficit’ (see Anderson and Pempek, 2005 for a review), contingent engagement helps children successfully transfer this knowledge. For instance, children who had difficulty finding a toy hidden in a room if they watched the toy being hidden in a pre-recorded video, were able to find the toy if the experimenter interacted with the child, over video, throughout the hiding episode (Troseth et al., 2006). Other work on knowledge transfer has examined infants’ ability to learn new words from screens and use them in real life, showing that by 24 months children learn the meanings of new words equally well in a live interaction and live video interaction, but not using pre-recorded non-interactive video (Roseberry et al., 2014). In other recent work, 104 parent–child dyads were videotaped using a touchscreen tablet to observe the supports and exchanges between parent and children ages 46–76 months. The results indicated that parents provided a great deal of support to their children while interacting with the touchscreen tablet including verbal, physical, and emotional support. The type of support offered did not differ as a function of parent gender or experience with mobile devices (users versus non-users) (Wood et al., 2016). Together, these results underscore the important role that a physically present and supportive adult may play in our results as well as in the broader literature. It is an open question whether the benefits of having some degree of social scaffolding during learning from interactive technology is similar to the benefits observed from social scaffolding when learning from more traditional ‘3D’ toys or reading books. Future work that compares in-person to digital learning should consider the potential influence of the presence or absence of such social factors during learning (see Lovato and Waxman, 2016 for review).

Finally, in addition to their contribution to the literature on children’s learning, our results offer much-needed empirical support for the validity of using interactive media for research purposes. We found that children ages 4 through 8 did not find it difficult to interact with the iPad. Moreover, given that children’s performance was comparable to an analogous ‘live’ experiment, this research suggests that interactive technology may be an appropriate method to collect data to test research questions on a range of topics within developmental science, not just for research evaluating the efficacy of children’s learning from interactive media. In fact, there were some clear benefits of using the interactive device over a live interaction for research purposes. For instance, the use of a pre-recorded tutorial insured that all participants experienced the exact same instructions using the same rate of speech and the same vocal intonations that is not possible when using live researchers. Computerized data collection methods also simplify data coding and data entry as responses and response times are automatically recorded, bypassing the need for more time-consuming coding of videotaped responses and the need for inter-rater reliability. Moreover, computerized data collection reduces the possibility of human error in inputting responses and eliminates the possibility of experimenter bias.

In sum, our results demonstrate that children 4 to 8 years of age learned factual information about animals equally well from an interactive iPad application as they did during face-to-face instruction. These results contribute much-needed data to the limited experimental evidence supporting the use of interactive media in children’s learning. These data may help alleviate the concerns of some parents and educators who believe that learning from interactive media is inferior to learning from real-life interactions. Of course, interactive media should never, and could never, replace the many benefits of real-world social interactions but can be used in moderation to supplement real-world learning. Indeed, as parents gain confidence in the educational value of interactive media they may change their assessment of its primary value from ‘entertainment purposes’ to ‘educational purposes’. Continued research in this field will have important implications for children’s learning and education, parents’ and educators’ attitudes about interactive technology, and research methodology.

## Author Contributions

SB and SG designed the study. KK designed the iPad app and wrote the first draft of the Introduction and Methods. PC developed the back-end data collection platform and assisted with application development. SG, TH, and VL assisted with data collection. VL analyzed the data and wrote the first draft of the Section “Results”. SB oversaw all aspects of the research. All authors contributed to the writing of the final manuscript.

## Conflict of Interest Statement

SB is the founder of the company, Applied Cognition Corp., that helped fund the development of the free iPad learning app used in the experiment presented in this manuscript. All the other authors declare that the research was conducted in the absence of any commercial or financial relationships that could be construed as a potential conflict of interest.

## Footnotes

1 Interestingly, one might expect that these parents would be reluctant to allow their child to use such devices, yet there is no correlation between negative parental attitudes about interactive technology and their reports of their child’s use of touch-screen devices (Wooldridge, 2016).

2 These data (i.e., children’s answers to “how many children will know [question]?”) were analyzed for separate research purposes and were not included in this manuscript.

3 The facts were repeated more often in the Face-to-Face condition than in the Interactive Media condition making the equivalent rates of learning across the two conditions possibly even more compelling. However, children’s requests to have the question or the fact repeated were quite rare occurring on less than 1% of all trials. The trials that children requested to have repeated most often corresponded with the answers: ‘Ruppell’s Vulture’ and ‘the Greater Wax Moth’ (tied for the most repeats), followed by the ‘Fairyfly’ and ‘Back of the neck’ (tied for second).
