# Supplementary material for: Children Can Learn New Facts Equally Well From Interactive Media Versus Face to Face Instruction
Source: Front Psychol. 2016 Oct 25;7:1603. doi: 10.3389/fpsyg.2016.01603 (PMC5078753; doi:10.3389/fpsyg.2016.01603)
Supplement: Supplementary file 1 [file Data_Sheet_1.docx]

**Appendix A**

**Detailed Description of the Wording used in the Demonstration Phase for the**

**Face-to-Face and Interactive Media Conditions**

Face-to-Face Condition: “Let me show you how to play. See this board? There’s no children in here, there’s a couple of children here, there’s some here, a lot here, and there’s a whole lot of children here! I am going to ask you some questions and you can show me how many children your age will know the right answer by pointing to one of these.” (A printout of the same board used in the Interactive Media condition was used, see Figure 1). “Let me show you how to play. A cow says moo. How many children your age will know that? That’s a really easy one. I think that a whole lot of children your age will know that one, so you’d point here!” The instructor pointed to the ‘a whole lot’ button on the five-point scale.

Then, the instructor said, “Let’s try another one. This is a holophonor. How many children your age will know that? That’s a really hard one. I just learned it myself. So no children your age will know that one. So you’d point here.” The instructor then proceeded to point to the ‘none’ button. Lastly, the instructor said, “A giraffe is the tallest animal. How many children your age will know that? That’s not really hard or easy. Some children will know that one, so you’d point here”. The instructor pointed to ‘some’ on the five-point scale. “Let’s try some other questions.”

Interactive Media Condition: “Let me show you how to play. See this board? There’s no children in here, there’s a couple of children here, there’s some here, a lot here, and there’s a whole lot of children here! I’m going to ask you some questions and you can show me how many children your age will know the right answer by tapping on one of these buttons. Make sure you’re tapping on the button and not the picture. Then, tap on the green arrow to go to the next question. Let me show you how to play. A cow says moo. How many children your age will know that? That’s a really easy one. I think that a whole lot of children your age will know that one, so you’d tap here!” A pointer finger directed children’s attention to ‘a whole lot’ and prompted them to tap on the button (see Figure 1, to view the scale). Then, the pointer finger directed the children’s attention to the green arrow and prompted them to tap there with a recording of, “Remember to tap on the green arrow”.

“Let’s try another one. This is a holophonor. How many children your age will know that? That’s a really hard one. I just learned it myself. So no children your age will know that one. So you’d tap here.” The pointer finger directed children’s attention to ‘none’ and prompted them to tap there, and then on the green arrow. “A giraffe is the tallest animal. How many children your age will know that? That’s not really hard or easy. Some children will know that one, so you’d tap here”. The pointer finger directed children’s attention to ‘some’ and prompted them to tap there, and then the green arrow.

“Let’s try some other questions. Remember, you can point to any of these when I ask you how many children will know the answer. There’s no children’s here, there’s a couple here, there’s some here, a lot here, and there’s a whole lot of children here.” A pointer finger indicated each button in sync with the audio. “Anytime you want to hear the question again, just tap on me, the Llama, and I’ll repeat it for you. Ok, let’s get started. Tap on the green arrow.”
